# Characterization of Chlorhexidine-Impregnated Cellulose-Based Hydrogel Films Intended for the Treatment of Periodontitis

**DOI:** 10.1155/2021/9853977

**Published:** 2021-09-15

**Authors:** Ola Tarawneh, Imad Hamadneh, Rawan Huwaitat, Ameen Rasheed Al-Assi, Abdulla El Madani

**Affiliations:** ^1^Department of Pharmacy, Faculty of Pharmacy, Al-Zaytoonah University of Jordan, P.O. Box 130, Amman 11733, Jordan; ^2^Chemistry Department, Faculty of Science, The University of Jordan, 11942 Amman, Jordan

## Abstract

Periodontitis comprises a chronic inflammation that is initiated by microbiota biofilm. If left untreated, periodontitis may lead to permanent tooth loss. Herein, we propose to design and improve a localized form of therapy comprising a chlorhexidine-impregnated hydrogel. Hydrogel films were prepared by varying the ratio between cellulose (MCC) and carboxymethylcellulose sodium (CMC) using the crosslinker epichlorohydrin (ECH). The hydrogel was loaded with chlorhexidine. Increasing the CMC ratio led to a reduction in the number of pores, an increase in their size, lower glass transition temperature (*T*_*g*_), decreased Young's modulus, and increased film stretching and affected the time of release. Bacterial and fungal zones of inhibition showed similar activity and were not affected by the CMC and MCC ratio. Hydrogels loaded with chlorhexidine prevented the growth of *S. oralis* and *C. albicans* microorganisms and may provide a promising local delivery system for treating periodontitis.

## 1. Introduction

Periodontitis is chronic multifactorial inflammatory diseases associated with mixed bacteria that colonize the tooth surface [1]. In the early stage, the disease may start as an inflammation of the gingiva, which could progress to the surrounding periodontal structures [2]. If left without therapy, periodontitis eventually leads to deterioration of connective tissue, tooth loss, and implant failure [3]. The main strategy in the management of periodontitis revolves around establishment of adequate infection control [1]. An essential approach in this management is motivated patient self-care, subgingival instrumentation, and the use of local/intrapocket antimicrobial delivery systems. Those local delivery systems are noninvasive and applied directly at the site of infection. Examples on drug delivery systems include hydrogel, oleogels, and bigels in which rheological properties are profoundly affected by the type of gel and subsequent adhesion [4], fibers which can be fabricated by several methods such as electrospun nanofibers [5], strips, and films which can be fabricated and tuned in many geometrical dimensions and shapes [6]. It is crucial for health care providers to consider pharmacological agents with conventional periodontal therapy through employing proper delivery systems to achieve best clinical results [7]. Hydrogels are three-dimensional insoluble macromolecules of hydrophilic polymer chains that are crosslinked chemically or physically [8]. Microbicide-loaded hydrogel films when inserted in the periodontal cavity demonstrate higher local concentrations of the active agent and reduce the risk of systemic side effects [9]. Chlorhexidine is an antiseptic agent that is employed in dental preparations due to its broad-spectrum activity against gram-positive and gram-negative bacteria and fungi [10]. Chlorhexidine was previously fabricated by Lim et al. in a dual delivery system for the treatment of periodontitis. The system was composed from a rapidly dissolving platform that led to complete drug release within 3 h, which implies the need of repeated administration of the films and eventually may lead to lack of patients' cooperation [11]. Moreover, the high rate of gingival fluid clearance leads to a rapid reduction in chlorhexidine therapeutic concentrations from the periodontal pocket and, thus, insufficient treatment effectiveness [10].

The objective of this study was to develop and employ antimicrobial hydrogel films for the treatment of periodontitis that can elute the drugs over a prolonged time to enhance patients' cooperation of drug administration. The hydrogels were prepared using a binary system based on CMC and cellulose to reinforce hydrogels' mechanical properties [8]. Carboxymethylcellulose sodium (CMC) was employed in preparing cellulose-based hydrogels [12]. CMC-hydrogel was developed to deliver the antiseptic agent chlorhexidine to dentin tubules [13]. The hydrogel films were freeze-dried to ensure the formation of pores. The films were loaded with the antiseptic agent chlorhexidine and were characterized by scanning the topographical features, conducting *in vitro* drug release, and evaluating the mechanical properties as well as antimicrobial activity of the hydrogel films. The exemplar species included herein were *Streptococcus oralis* and *Candida albicans*.

## 2. Materials and Methods

### 2.1. Chemicals and Reagents

Microcrystalline cellulose (MCC) powder was purchased from Sigma Aldrich, USA, having an average particle size of 51 *μ*m, and its density is 0.6 g/mL; carboxymethylcellulose sodium salt (Na-CMC) was resourced from Sigma Aldrich (USA) with a molecular weight of 90,000 g/mol; epichlorohydrin (ECH) (1.18 g/mL) with purity ≥ 99% was purchased from Sigma Aldrich (USA); chlorhexidine purity greater than 99.5% CAS number 55-65-1 was purchased from Sigma Aldrich (USA); and sodium hydroxide (NaOH) ACS reagent with purity of ≥98% pellet was purchased from Sigma Aldrich. Phosphate buffer solution (PBS, pH 7.4) was obtained from Sigma Aldrich.

### 2.2. Preparation of Hydrogel

Films were prepared using crosslinking reaction as described by Chang and colleagues with modification [14]. For F1 (Table 1), 4 g of MCC was dispersed in 100 mL of water. Then, 4 g of CMC was added to the resultant dispersion system with continuous stirring. 16 mL of the crosslinker, EPH, was added to the resultant mixture and stirred for 2 h at 30°C till gel was obtained. The gel was washed with water, poured into a Petri dish, and stored at -20°C for 12 h. Afterward, the films were freeze-dried for 72 h at -72°C. The process was repeated using variable ratios as explained in Table 1.

### 2.3. Film Morphological Characterization

Surface topography of the dried films was investigated by Scanning Electron Microscope (SEM) using VERSA 3D from FEI (Thermo Fisher Scientific, USA), as previously described by our group [15]. The cross-section of the films was examined without coating. Micrographs were captured using a 2.00 kV acceleration voltage of the secondary electron (SE) mode.

### 2.4. Determination of the Glass Transition Temperature

The glass transition temperature (*T*_*g*_) and the thermal properties of the dried film formulations were tested using Q8000 DMTA (TA, USA). The temperature scan was performed in the temperature range from zero to 140°C at a heating rate of 3°C/min in tensile mode at an oscillatory frequency of 1 Hz. The films were cut into rectangular shapes (average dimensions of 3 cm length, 0.5 cm width, and 0.11 cm thickness). The *T*_*g*_ was determined from the peak of the tan *δ* lines and presented as a mean of three replicates [16].

### 2.5. Mechanical Properties

The tensile analyses of the dried films were performed using a Stable Micro Systems TA-XT2 texture analyzer (Godalming, Surrey, UK). Five replicate samples of each film (30 × 5 mm) were clamped between the static grip which is the lower grip and the upper moveable grip clamps of the TA-XT2 texture analyzer, ensuring that the length of the films under stress was constant (20 mm). The upper clamp was raised at a fixed rate (0.5 mm s^−1^) until fracture of the films occurred. From the resultant stress-strain plot, the ultimate tensile strength, percentage elongation at break point, and Young's modulus were calculated as reported in literature [15].

### 2.6. Drug Loading and In Vitro Release Kinetics

Chlorhexidine solution (10 mL of 0.15% *g*/*w*) was prepared, and the dried films were soaked for three days in the solution in order to load the drug. After reswelling equilibrium was reached, the drug-loaded hydrogels were transferred into Duran bottles containing 20 mL of prewarmed phosphate buffer solution (PBS pH = 7.4) to determine the release of the chlorhexidine at 37°C. At predetermined time intervals (0.5, 1, 4, 24, 48, 72, 120, 144, 168, 216, 288, and 312 h), PBS was withdrawn and replaced with a fresh 20 mL prewarmed buffer to ensure sink condition. The mass of cumulative released chlorhexidine was analyzed using ultraviolet spectroscopy (UV-visible spectrophotometer, Varian Cary, USA) at *λ*_max_ 295 nm. The released chlorhexidine was calculated concerning a previously standardized calibration curve (where *r*^2^ was greater than 0.99). The percentage of drug released was then plotted against time. The release mechanism was studied using the general law power of the Korsmeyer-Peppas equation: Mt/M∞ = *ktn* where Mt/M∞ is the fractional solute release, *t* is the release time, *k* is a kinetic constant, and *n* is the release exponent. The value of *n* describes the mechanism involved in drug release, where a value ~0.5 describes a Fickian diffusion, and a value in the range of 0.5-1 is said to have an anomalous (non-Fickian) diffusion [17].

### 2.7. Antimicrobial Activity

Antimicrobial activity of chlorhexidine-loaded films was tested against *Streptococcus oralis* (ATCC 6249) and *Candida albicans* (ATCC 10231) according to the Kirby-Bauer method [18]. An inoculum from overnight cultures was optically adjusted to obtain 1 × 10^8^ CFU/mL. Müller-Hinton solid agar was inoculated with 100 *μ*L bacterial or candida cultures in Petri dishes followed by placement of formulation-impregnated discs (5 mm diameter, *n* = 5) on the cultured agar plates. After 15 min, the *S. oralis*-cultured agar plates and *C. albicans*-cultured agar plates were incubated at 37°C for 48 h. Blank films without chlorhexidine were also tested for their antimicrobial activity. Filter paper discs saturated with chlorhexidine (5 mm diameter) were tested as a control.

### 2.8. Statistical Analysis

Statistical analysis of results was performed using GraphPad Prism 8 using one-way ANOVA followed by the Tukey-Kramer post hoc test. Samples were expressed as mean ± standard deviation (SD). The statistical value of *p* < 0.05 was denoted as significant.

## 3. Results and Discussion

### 3.1. Film Morphology

**Figure 1:**
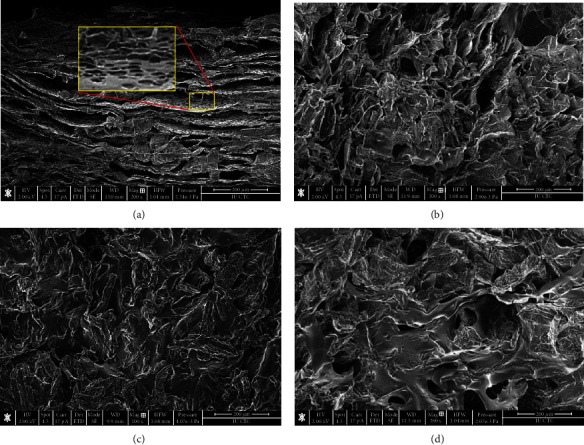
SEM micrographs of the topological surface of the dried hydrogels of variable CMC : MCC ratios (wt : wt): (a) F1 (4.0 : 4.0), (b) F2 (4.5 : 3.5), (c) F3 (5.0 : 3.0), and (d) F4 (5.5 : 2.5). Scale bar = 200 *μ*m.

The microstructure of the prepared films is shown in Figure 1. All samples exhibited microporous architecture; Figure 1(a) illustrates highly oriented porous sheets of cellulose CMC which appear to be layered above each other. Pores are distributed all over the surface with an average size of 7-15 *μ*m in each layer. The CMC serves as a linker between the layers; hence, as the CMC ratio increases, linkers of the layers become wider, creating pockets between the layers with the average pore size of 90-200 *μ*m (Figure 1(b)). Furthermore, the addition of CMC increases the thickness of the walls and reduces the number of the pores, suggesting that CMC's addition might enhance the polymer's flexibility and surface smoothness (Figures 1(c) and 1(d)) which was suuported by the results of mechanical tests. The effect of CMC on the number of pores and thickness of walls could be attributed to the negative charge of the carboxylates that would initiate an electrostatic repulsion which may expand the configurations of the copolymer chains. This is in good agreement with Reference [14]. The films have a longer leaching time which supports the in vitro release results.

### 3.2. Determination of the Glass Transition Temperature

**Figure 2:**
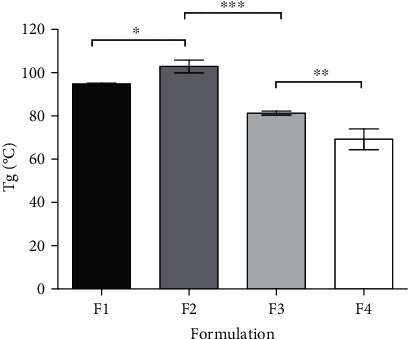
*T*_*g*_ of the hydrogel dried films detected by DMTA. F1 represents film composed of 4.0 : 4.0, F2 composed of 4.5 : 3.5, F3 composed of 5.0 : 3.0, and F4 composed of 5.5 : 2.5 CMC : MCC. Values shown presenting mean ± SD, *n* = 3. ^∗^*p* < 0.05, ^∗∗^*p* < 0.01, and ^∗∗∗^*p* < 0.001: significant differences between formulations.

*T*_*g*_ results of dried films are displayed in Figure 2. Generally, the observations indicate that the increase in the CMC ratio resulted in reduced *T*_*g*_ values. F4 films containing the higher ratio of CMC (5.5 : 2.5 CMC : MCC) showed a significant reduction in *T*_*g*_, 69.21 ± 4.80°C, compared to F1, F2, and F3 where the *T*_*g*_ values were 94.91 ± 0.26, 102.88 ± 2.98, and 81.32 ± 0.95°C, respectively. The addition of CMC to MCC showed a marked decrease in the thermal pattern of cellulose that has a *T*_*g*_ range of 200-250°C [19] while *T*_*g*_ of CMC films is approximately 79°C. Low *T*_*g*_ of CMC denotes the poor rigidity of the polymer which explains the decrease of *T*_*g*_ temperature with elevation in the CMC content [20]. The use of an equal amount of CMC and MCC led to strong interactions. The *T*_*g*_ results are consistent with micrograph images from SEM, where increasing the wall thickness and pore size would increase the void volume in the film and thus enhance its flexibility and lower the *T*_*g*_.

### 3.3. Mechanical Properties

**Figure 3:**
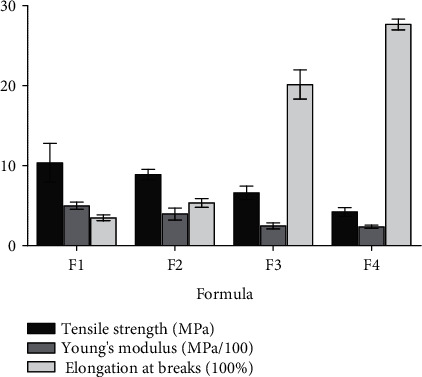
Tensile strength, Young's modulus, and elongation at break of films at different CMC : MCC ratios. F1 represents film composed of 4.0 : 4.0, F2 composed of 4.5 : 3.5, F3 composed of 5.0 : 3.0, and F4 composed of 5.5 : 2.5 CMC : MCC. Values shown are expressed as mean ± SD of five replicates.

The mechanical properties are crucial elements regarding the evaluation of the microstructure in biomaterials [21]. Glassy brittle biomaterials are susceptible to cracking providing a suitable environment for bacterial adherence [22]. The investigation of the effect of the CMC ratio on the tensile strength, tensile modulus, and percentage of elongation of CMC/MCC films is displayed in Figure 3. Tensile strength and Young's modulus of films decrease upon lowering CMC ratio while the elongation at break of the films increases. When the CMC ratio increased from 4 to 5.5 g, the tensile strength decreased from 10.3 to 4.2 MPa and the tensile modulus significantly decreased from 498.4 to 235.7 MPa (*p* < 0.001). On the other hand, the elasticity indicated by percentage elongation is significantly increased from 3.6% to 27.6% (*p* < 0.001). However, the tensile strength exhibited no significant difference between the formulations (*p* > 0.05) at different ratios. It is not surprising that the increase in CMC content lowered the rigidity and plasticity of the films and increased their flexibility as CMC showed poor mechanical properties manifested by the low *T*_*g*_ temperature [20]. In view of the aforementioned results, MCC acted as a backbone agent for the films providing strength to the formulated films as observed by the high tensile strength and Young's modulus values.

### 3.4. Drug Loading and In Vitro Release Kinetics

**Figure 4:**
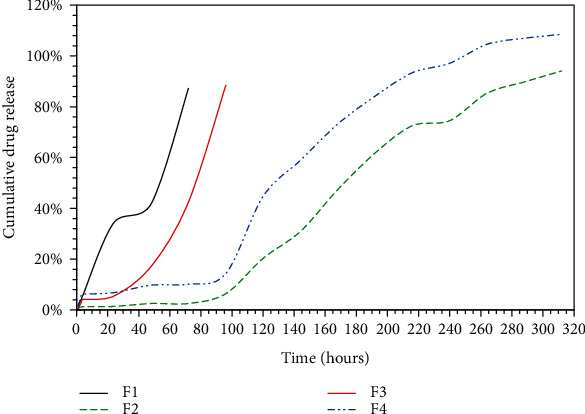
*In vitro* cumulative release profiles of chlorhexidine from hydrogel films of variable MCC : CMC content ratios in PBS (pH 7.4) at 37°C. F1 represents films composed of 4.0 : 4.0, F2 composed of 4.5.0 : 3.5, F3 composed of 5.0 : 3.0, and F4 composed of 5.5 : 2.5 CMC : MCC. Values are expressed as mean ± SD of three replicates.

The drug loading was 15 mg/10 mL for each film, soaked for equilibrium for 3 days. Then, the film was removed and washed. The absorbance of the remaining solution was measured, and the amount was calculated by referral to the standardized calibration curve. To obtain the actual amount in each film, we subtracted the remaining amount of chlorhexidine in the remaining solution from the total amount of chlorhexidine. Therefore, F2 showed the highest loading capacity with 6.3 mg, then F4 with 8.8 mg. Drug loading was consistent with the SEM micrograph where F2 demonstrated high porosity, whereas F4 displayed the greatest pore size. The release studies were conducted to study the drug release rate of the CMC/MCC hydrogels in PBS pH 7.4, at 37°C for 13 days. The *in vitro* cumulative percentage release profiles of chlorhexidine from hydrogels are shown in Figure 4. The cumulative percentage release was complete in all formulations. The release of chlorhexidine was affected by the ratio of CMC and MCC. Initially, the observed release was less than 2% for the four tested formulations. F1 and F3 reached 90% drug release within 72 and 96 h, respectively. However, F2 and F4 films exhibited a notable slower release where less than 10% of chlorhexidine was released within 96 hours. However, both formulations showed extended controlled release with respect to time, where the release from F2 reached 94.08 ± 2.73%, and F4 exhibited a complete drug release of 108.50 ± 6.96% after 13 days (312 h). The different pattern of chlorhexidine release from the investigated formulations was consistent with the porosity characteristics obtained from the film morphology studies. The F2 films, which displayed the highest porosity, and F4, which showed the largest pore size, demonstrated a greater chlorhexidine cumulative percentage of release compared with the F1 and F2 films. Pore size increased upon elevating the CMC content, leading to increased chlorhexidine uptake into hydrogels during the reswelling of dried films [22]. As a result, F2 and F4 films exhibited a faster release of chlorhexidine, which was trapped in the pores in the presence of water or physiological solutions [23]. The observed slow release in F3 films can be explained by the decreased number of pore content, which led to a low concentration of chlorhexidine loading and fast drug release. The results revealed the promising coverage of antimicrobial concentration for the medical devices, applying the release exponent (*n*) from the Korsmeyer-Peppas equation of the general power expression [18], for a release percentage less than 60 and not less than 4 determination points. The release exponent was less than 0.5 in F2 and F4 indicating that the release mechanism was governed by Fickian diffusion. However, for F 1 and F3, the *n* value was greater than 1 indicating that the release was controlled by swelling. Similar devices composed from CMC and MCC hydrogel were loaded with bovine serum albumin. The release was for oral delivery and was complete within 40 h which is optimum for this route. However, the differences in the method of measuring drug release are due to different applications.

### 3.5. Antimicrobial Activity

The Kirby-Bauer method was employed to assess the four prepared formulations' ability to inhibit the growth of *S. oralis and C. albicans*. Both tested species are commonly observed in periodontitis. The tested films were compared against similar composition but void of chlorhexidine. The zones of inhibition values after 48 h are shown in Table 2. The chlorhexidine-free films did not show an inhibition zone; therefore, the loaded films' inhibition zones are due to the release of chlorhexidine. Furthermore, the mean inhibition zone in the loaded films was in the range from 14.4 to15.8 mm against *S. oralis* and 15-5.3 mm against *C. albicans*. The zone of inhibition diameter slightly decreased with decreasing CMC content with the observed inhibition zone against *C. albicans* being lower than the mean values observed against *S. oralis*. However, the antimicrobial activities of different ratios of the hydrogel films were similar to the control which was void of chlorhexidine (*p* > 0.05). F1 films showed the highest inhibition zone after 48 h compared to the others with a zone of inhibition 15.8 ± 0.5 mm and 15.3 ± 0.5 mm against *S. oralis* and *C. albicans*, respectively. The results are coherent with drug release profiles where F1 displayed a higher percentage cumulative release of around 42% after 48 h, while the cumulative percentage release of F2 and F4 films was less than 10%. Hence, the microorganisms under investigation were exposed to higher concentrations of chlorhexidine when incubated with F1 films. Chlorhexidine is an antibacterial and antifungal antiseptic agent commonly used to treat periodontal diseases [22]. It prevents microbial growth by disrupting bacterial membranes and targeting membrane-bound enzymes, followed by leakage of cytoplasmic components [24]. The slow release of chlorhexidine from the prepared films would suggest that this local drug delivery system would serve as a promising delivery system for the treatment of periodontitis. The film can be inserted in the infected pocket and deliver the drug locally over a satisfying period that could be two weeks, as seen in F2 and F4. In this way, the patient may not require systemic antibiotics administration and be exposed to the risk of developing antimicrobial resistance by the local microorganisms in the oral cavity.

## 4. Conclusions

Four hydrogel films were successfully produced from CMC and MCC with different ratios in the ECH's presence as a crosslinker. The dried hydrogel films displayed favorable thermal and mechanical properties and exhibited extended drug release at least three days presented by F1 and up to 13 days demonstrated by the F3 and F4 films, suggesting that the employed ratio of CMC could tune the release time. Hydrogels loaded with chlorhexidine prevent the growth of *S. oralis* and *C.* a*lbicans* microorganisms. F2 and F4 may be a promising local delivery system for treating periodontitis, where the release was extended over two weeks.

## Figures and Tables

**Table 1:** Chemical composition of the CMC : MCC hydrogels.

Formula	CMC (g)	MCC (g)	ECH (mL)
F1	4.0	4.0	16
F2	4.5	3.5	16
F3	5.0	3.0	16
F4	5.5	2.5	16

**Table 2:** Zone of inhibition of CMC/MCC films against tested microorganisms.

Formula	Zone of inhibition (mm)	
	*S. oralis*	*C. albicans*
F1	15.8 ± 0.5	15.3 ± 0.5
F2	14.5 ± 0.5	15.2 ± 0.4
F3	14.5 ± 0.6	15.0 ± 0.0
F4	14.4 ± 0.5	15.0 ± 0.0
Control	15.5 ± 0.5	15.5 ± 0.5

## Data Availability

All data are described in the manuscript.

## References

[1] Suvan J., Leira Y., Moreno Sancho F. M., Graziani F., Derks J., Tomasi C. (2020). Subgingival instrumentation for treatment of periodontitis. a systematic review. *Journal of Clinical Periodontology*.

[2] Tong X., Qi X., Mao R. (2020). Construction of functional curdlan hydrogels with bio-inspired polydopamine for synergistic periodontal antibacterial therapeutics. *Carbohydrate Polymers*.

[3] Heitz-Mayfield L. J. A., Salvi G. E. (2018). Peri-implant mucositis. *Journal of Periodontolgy*.

[4] Hamed R., AbuRezeq A., Tarawneh O. (2018). Development of hydrogels, oleogels, and bigels as local drug delivery systems for periodontitis. *Drug Development and Industrial Pharmacy*.

[5] Chou S. F., Carson D., Woodrow K. A. (2015). Current strategies for sustaining drug release from electrospun nanofibers. *Journal of Controlled Release*.

[6] Castro P. M., Fonte P., Sousa F., Madureira A. R., Sarmento B., Pintado M. E. (2015). Oral films as breakthrough tools for oral delivery of proteins/peptides. *Journal of Controlled Release*.

[7] Rajeshwari H. R., Dhamecha D., Jagwani S. (2019). Local drug delivery systems in the management of periodontitis: a scientific review. *Journal of Controlled Release*.

[8] Chang C., Lue A., Zhang L. (2008). Effects of crosslinking methods on structure and properties of cellulose/PVA hydrogels. *Macromolecular Chemistry and Physics*.

[9] al Othman Z. A., Alam M. M., Naushad M., Inamuddin, Khan M. F. (2013). Inorganic nanoparticles and nanomaterials based on titanium (Ti): applications in medicine. *Materials Science Forum*.

[10] Zhao H., Hu J., Zhao L. (2020). Adjunctive subgingival application of chlorhexidine gel in nonsurgical periodontal treatment for chronic periodontitis: a systematic review and meta-analysis. *BMC Oral Health*.

[11] Lim S. Y., Dafydd M., Ong J. (2020). Mucoadhesive thin films for the simultaneous delivery of microbicide and anti- inflammatory drugs in the treatment of periodontal diseases. *International Journal of Pharmaceutics*.

[12] Sannino A., Madaghiele M., Lionetto M. G., Schettino T., Maffezzoli A. (2006). A cellulose-based hydrogel as a potential bulking agent for hypocaloric diets: an in vitro biocompatibility study on rat intestine. *Journal of Applied Polymer Sciences*.

[13] Kovtun A., Kozlova D., Ganesan K. (2012). Chlorhexidine-loaded calcium phosphate nanoparticles for dental maintenance treatment: combination of mineralising and antibacterial effects. *RSC Advances*.

[14] Chang C., Duan B., Cai J., Zhang L. (2010). Superabsorbent hydrogels based on cellulose for smart swelling and controllable delivery. *European Polymer Journal*.

[15] Tarawneh A., al-Ass A. R., Hamed R. (2021). Development and characterization of k-carrageenan platforms as periodontal intra-pocket films. *Tropical Journal of Pharmaceutical Research*.

[16] Mahmoud N. N., Qabooq H., Alsotari S. (2021). Quercetin-gold nanorods incorporated into nanofibers: development, optimization and cytotoxicity. *RSC Advances*.

[17] Korsmeyer R. W., Gurny R., Doelker E., Buri P., Peppas N. A. (1983). Mechanisms of solute release from porous hydrophilic polymers. *International Journal of Pharmaceutics*.

[18] Abral H., Ariksa J., Mahardika M. (2020). Transparent and antimicrobial cellulose film from ginger nanofiber. *Food Hydrocolloids*.

[19] Szcześniak L., Rachocki A., Tritt-Goc J. (2008). Glass transition temperature and thermal decomposition of cellulose powder. *Cellulose*.

[20] Misra S. K., Valappil S. P., Roy I., Boccaccini A. R. (2006). Polyhydroxyalkanoate (PHA)/inorganic phase composites for tissue engineering applications. *Biomacromolecules*.

[21] Qiu K., Netravali A. N. (2012). Fabrication and characterization of biodegradable composites based on microfibrillated cellulose and polyvinyl alcohol. *Composites Science and Technology*.

[22] Inoue B. S., Streit S., dos Santos Schneider A. L., Meier M. M. (2020). Bioactive bacterial cellulose membrane with prolonged release of chlorhexidine for dental medical application. *International Journal of Biological Macromolecules*.

[23] Treesuppharat W., Rojanapanthu P., Siangsanoh C., Manuspiya H., Ummartyotin S. (2017). Synthesis and characterization of bacterial cellulose and gelatin-based hydrogel composites for drug-delivery systems. *Biotechnology Reports*.

[24] Russell A. D., Day M. J. (1993). Antibacterial activity of chlorhexidine. *Journal of Hospital Infection*.

